# Self-worth systems in aging societies: a narrative review and conceptual framework from self-esteem to healthy longevity

**DOI:** 10.3389/fpsyg.2026.1879051

**Published:** 2026-07-10

**Authors:** Jiaxin Cai, Lei Yu, Huixia Huang, Yanlan Guo, Fengxue Shi

**Affiliations:** 1 Hunan Institute of Science and Technology, Yueyang, Hunan, China; 2 College of Physical Education, Hebei Normal University, Shijiazhuang, Hebei, China; 3 School of Physical Education and Training, Capital University of Physical Education and Sports, Beijing, China; 4 School of Physical Education, North University of China, Taiyuan, Shanxi, China; 5 Binhu Road Primary School of Nanning, Nanning, Guangxi, China

**Keywords:** ageism, aging, autonomy, digital exclusion, dignity, healthy longevity, loneliness, long-term care

## Abstract

Aging societies face not only challenges of health care, dependency and disease burden, but also a psychosocial challenge: how to preserve older adults’ sense that their lives, choices, relationships and contributions continue to matter. This narrative review and conceptual framework examines later-life self-worth as a multilevel system shaped by personal, relational, institutional, cultural and policy conditions. We distinguish self-worth from global trait self-esteem and define later-life self-worth as the perceived significance of one’s life, agency, dignity, contribution and social visibility under changing conditions of capacity and dependency. The proposed framework organizes self-worth around six dimensions: autonomy, dignity, mattering, reciprocity, role continuity and social visibility. We synthesize evidence on social connection, loneliness, reciprocity, retirement, ageism, functional decline, long-term care, digital exclusion and cultural disruption, while also identifying areas where evidence is indirect, heterogeneous or primarily correlational. We argue that self-worth may influence healthy aging through psychological, behavioral, social, cognitive and stress-related biological pathways, but that causal claims remain limited and require longitudinal, experimental and mixed-method research. Finally, we discuss strategies for preserving self-worth, including autonomy-supportive environments, dignity-centered care, social prescribing, intergenerational programs and digital inclusion. Healthy longevity should therefore be understood not only as survival, function or disease control, but also as the preservation of a life that is recognized, chosen, connected and meaningful.

## 1 Introduction

The aging population is frequently viewed as a challenge concerning health care, pensions, and dependency. Yet aging societies also face a deeper social-psychological problem: how to preserve older adults’ sense that they remain valued, agentic and consequential. Contemporary organizations frequently prioritize efficiency, self-reliance, rapidity, and digital skills. As individuals progress through retirement, face bereavement, chronic illnesses, declining functionality, dependency on care, and exclusion from technology, they might repeatedly perceive themselves as less useful, capable, or socially visible. Research on self-esteem serves as a crucial foundation. Longitudinal evidence suggests that self-esteem generally rises from childhood into adulthood, reaches relatively high levels in midlife or early later life, and declines more clearly in advanced old age (Orth et al., 2018; Robins and Trzesniewski, 2005). However, this pattern should not be interpreted as an entirely personal journey. Sociometer theory proposes that self-esteem functions as an internal monitor of perceived relational value (Leary et al., 1995; Leary, 2005). Consequently, the decline in self-esteem among the elderly might be partly due to shifts in the social setting where they are recognized, necessary, and respected.

This Review argues that self-esteem is too narrow to capture these dynamics. We propose a broader concept: self-worth systems in later life. Self-worth is the feeling that one’s life, choices, contributions, and existence remain significant. The organization is based on autonomy, dignity, importance, reciprocity, role continuity, and social visibility. These dimensions are influenced not just by psychological traits but also by families, communities, care institutions, digital infrastructures, cultural norms, and public policies. This framework aligns with major theories of aging and health. Healthy aging emphasizes functional ability as the interaction between intrinsic capacity and enabling environments (Beard et al., 2016). According to self-determination theory, autonomy, competence, and relatedness are fundamental psychological needs (Ryan and Deci, 2000). According to life-span motivational theories, aging is associated with the adaptation of goals, control, and emotional significance as opportunities change (Carstensen, 2021; Heckhausen et al., 2010). These viewpoints suggest that self-worth in later life is actively managed by aligning personal abilities, social connections, and environmental opportunities.

Self-worth is also shaped by social and structural conditions. Stronger social relationships are associated with lower mortality risk, and social connection is increasingly recognized as a public-health issue (Holt-Lunstad et al., 2010; Umberson and Montez, 2010; Holt-Lunstad, 2022). Increased mortality and poorer health outcomes are linked to loneliness and social isolation (Holt-Lunstad et al., 2015; Ong et al., 2016; Cacioppo and Hawkley, 2009). Another significant danger of ageism is that age stereotypes can be internalized throughout life, and it is linked to negative health effects in different contexts (Levy, 2009; Chang et al., 2020; Mikton et al., 2021). The loss of autonomy, privacy, participation, or dignity due to care dependency, long-term care, and digital exclusion can further diminish self-esteem (Chochinov, 2002; Lothian and Philp, 2001; Kim et al., 2020; Kim et al., 2014; Zarghami et al., 2025). Self-worth is important for health because it can coordinate various pathways. Low self-esteem predicts later depression and anxiety (Boyle et al., 2010), while purpose, social participation and meaningful engagement are associated with healthier behaviors, preventive care, cognitive outcomes and well-being (McEwen, 1998; Slavich and Irwin, 2014; van Loon et al., 2021; Fuseini et al., 2022; Wulandari and Rochmawati, 2024; Percival et al., 2022; Pescheny et al., 2020). Ongoing threats to self-worth may be associated with stress-related, inflammatory, behavioral and social-isolation pathways, although direct evidence for self-worth as a distinct biological mechanism remains limited (Bild and Pachana, 2022; Rapo et al., 2023; Peters et al., 2021; Canedo-García et al., 2017; Webster et al., 2024; Oh et al., 2021). Thus, self-worth should be seen as a regulatory link between social experiences and healthy aging.

This article is a narrative review and conceptual framework rather than a systematic review or meta-analysis. Its aim is not to estimate the pooled effect of self-worth on health outcomes, but to synthesize major theoretical and empirical strands that explain how older adults’ perceived worth is produced, threatened and preserved across personal, relational and structural contexts. We first distinguish self-esteem from broader later-life self-worth, then describe the proposed self-worth systems framework, examine social architectures and threats to self-worth, evaluate pathways linking self-worth to healthy longevity, and discuss intervention, research and policy implications. Throughout the review, we distinguish relatively well-established evidence from areas where the evidence remains indirect, heterogeneous or theoretically inferential.

## 2 Review approach

This review used a narrative and conceptual approach. We did not aim to conduct a systematic review, meta-analysis or scoping review. Instead, we sought to integrate evidence and theory from psychology, gerontology, public health, long-term care, sociology, ethics and digital inclusion in order to develop a framework of later-life self-worth systems. Literature was identified through iterative searches of PubMed, Web of Science, PsycINFO, Google Scholar and reference lists of key articles. Search terms included combinations of “self-worth,” “self-esteem,” “mattering,” “dignity,” “autonomy,” “aging,” “older adults,” “healthy aging,” “successful aging,” “loneliness,” “social isolation,” “ageism,” “retirement,” “long-term care,” “digital exclusion,” “social prescribing,” and “intergenerational programs.” Priority was given to meta-analyses, systematic reviews, longitudinal studies, influential theoretical papers and studies directly relevant to older adults.

Because the aim was conceptual synthesis rather than exhaustive evidence mapping, no formal risk-of-bias assessment or quantitative pooling was conducted. This limitation is addressed in the Discussion. To strengthen analytical transparency, the review distinguishes between direct evidence on self-worth or closely related constructs, indirect evidence from adjacent constructs such as purpose, dignity or social connectedness, and theoretical inference where evidence remains limited.

## 3 Findings

### 3.1 From self-esteem to self-worth systems in later life

#### 3.1.1 Beyond trait self-esteem

Self-esteem has usually been considered a comprehensive assessment of oneself, typically viewed as a stable personal trait throughout one’s life. Research over time and across different ages indicates that self-esteem typically increases from childhood to adulthood, peaks during midlife or early senior years, and then noticeably decreases in very old age (Orth et al., 2018; Robins and Trzesniewski, 2005). However, this typical pattern should not be seen as proof that self-esteem in older age is simply a fixed psychological trait. Instead, the drop in self-esteem later in life might indicate a more extensive restructuring of the social, functional, and institutional contexts that allow older adults to perceive themselves as capable, appreciated, and socially acknowledged.

One significant drawback of traditional self-esteem studies is that they frequently reduce varied late-life experiences to a single overall score. Retirement, losing a spouse, chronic illness, declining functionality, limited mobility, reliance on care, and social isolation do not just ‘lower self-esteem’; they change the environment where self-worth is created, acknowledged, and maintained. From this perspective, self-esteem is more accurately seen as a measurable signal that emerges from a wider self-worth system, rather than the entire system itself.

This reinterpretation aligns with sociometer theory, suggesting that self-esteem acts as an internal gauge of perceived social value instead of being a standalone self-perception (Leary et al., 1995; Leary, 2005). Older adults’ self-esteem is influenced by social settings that express inclusion, respect, usefulness, and recognition from this angle. The important inquiry is not solely about whether an older adult reports high or low self-esteem, but if their social circle continues to affirm their necessity, consultation, respect, and meaningful presence.

The self-worth viewpoint does not presume that increasing self-esteem scores is always the primary objective. Research on contingencies of self-worth shows that self-evaluation can become unstable when it depends heavily on achievement, external approval, ability or social comparison (Crocker and Wolfe, 2001). This is especially relevant in later life, when previously reliable sources of value, including occupational productivity, physical independence, financial control and family caregiving roles, may become less available. In this review, self-esteem is treated as a measurable signal of global self-evaluation, whereas self-worth refers to the broader conditions under which older adults experience themselves as significant, respected, agentic and socially consequential. Thus, the framework shifts attention from asking whether older adults report high or low self-esteem to asking how families, care systems, communities, technologies and policies create or erode the conditions for valued personhood (Table 1).

**Table 1:** Key constructs in the later-life self-worth systems framework.

Construct	Working definition in this review	Relation to self-worth systems
Self-esteem	A global evaluative attitude toward oneself, often measured as high or low self-regard.	Treated as a measurable signal, but not the whole system.
Self-worth	The perceived significance of one’s life, choices, contributions, dignity and social visibility.	Central organizing construct of the framework.
Dignity	Recognition of the older person as having inherent worth, biography, moral standing and bodily privacy.	A core dimension and ethical condition of self-worth.
Autonomy	Meaningful agency over decisions affecting daily life, including supported agency under dependency.	A core dimension; not equivalent to complete independence.
Mattering	The sense that one is important to others and has an effect on their lives.	A relational mechanism through which self-worth is calibrated.
Reciprocity	The ability to both receive and provide care, support, knowledge or contribution.	Protects self-worth by preventing one-sided dependency.
Role continuity	Preservation or reconstruction of meaningful roles across retirement, illness, relocation or care transitions.	Maintains identity continuity under changing capacity.
Social visibility	Being seen, heard, consulted and represented in family, community, institutional and digital settings.	Protects against invisibility, exclusion and symbolic devaluation.

#### 3.1.2 Self-worth, dignity and autonomy

The concept of self-worth in older age should be separated from the more narrowly defined psychometric notion of self-esteem. Self-esteem involves a person’s general judgment of themselves, whereas self-worth is the deeper belief that one’s life, preferences, contributions, and presence are important. In later years, a person’s sense of value is closely linked to their dignity and independence. Dignity relates to treating a person as having inherent worth, a unique background, and moral significance, while autonomy pertains to maintaining significant control over decisions affecting daily life.

This distinction is vital since many challenges to self-worth in older age are not solely due to self-evaluation. They emerge from social norms that depict the elderly as dependent, risky, costly, or a burden. Dignity-conserving care highlights that making patients feel appreciated is not an extra aspect of care, but a core part of compassionate clinical practice (Chochinov, 2002). The dignity of older individuals is jeopardized when their privacy is breached, decisions are made without consulting them, functional limitations are perceived as personal failures, or institutional practices prioritize efficiency over personal identity (Lothian and Philp, 2001; Clancy et al., 2021). Research from long-term care environments indicates that dignity is influenced by factors such as autonomy, privacy, family participation, physical limitations, and caregiving methods, rather than solely by psychological traits (Dong et al., 2021).

Autonomy is just as crucial to self-esteem. According to self-determination theory, autonomy, competence, and relatedness are fundamental psychological needs that enhance motivation and well-being throughout life (Ryan and Deci, 2000). In later years, autonomy should not be mistaken for total independence, as many seniors experience chronic illnesses, mobility issues, or require care. If successful aging is defined solely by independence, then dependency becomes something to be ashamed of. A better framework is supported autonomy, which maintains choice, preference, agency, and participation even when practical help is required. This perspective is consistent with the healthy aging model, which highlights functional ability as the result of the interaction between intrinsic capacity and supportive environments (Beard et al., 2016).

The suggestion is that maintaining self-esteem involves not denying dependency, but ensuring it does not lead to devaluation. An elderly individual who needs support with bathing, medications, digital services, or medical visits can maintain a high sense of self-esteem if the help provided respects their privacy, autonomy, mutual exchange, and personal identity. On the other hand, providing technically sufficient care can diminish self-esteem if it eliminates decision-making, overlooks personal history, treats the individual as a task, or implies that their preferences are bothersome. Thus, the primary psychological question is not about older adults being entirely independent, but about their recognition as agents in an interdependent context.

#### 3.1.3 A dynamic model of self-worth regulation

A self-worth systems framework depends on having a dynamic model. Older adulthood is characterized by a series of transitions that frequently change the equilibrium between autonomy, recognition, and dependence. Retirement can break productivity identity, bereavement can break relational validation, chronic illness can restrict activity space, moving or institutional care can break place and role continuity, digitalized services can make everyday tasks feel incompetent or excluded. Every change adjusts self-esteem by altering the responses to three fundamental questions: Can I still take action? Am I still valued by others? Do people still treat me with dignity?

This model is based on theories of motivation across the lifespan. According to socioemotional selectivity theory, the way people perceive time affects their motivational priorities, with emotionally significant goals gaining importance as they grow older (Carstensen, 2021). The motivational theory of life-span development highlights how people adjust to age-related opportunities and limitations by managing control, letting go of unreachable goals, and focusing on different avenues of action (Heckhausen et al., 2010). These models suggest that self-worth in older age is not merely lost or maintained; it is actively restructured based on evolving abilities, objectives, and social environments. Three interacting regulatory layers are part of the proposed model. The initial layer involves personal aspects such as self-perceptions of aging, perceived abilities, confidence in one’s body, outlook on future time, and sense of meaning. The relational layer, which is the second, covers elements such as importance, mutuality, belongingness, family roles, the quality of friendships, and social visibility. The third layer involves structures like retirement systems, care facilities, ageism, digital infrastructure, neighborhood planning, and cultural stories about aging. Self-worth is managed through interactions between these layers. For example, functional decline may reduce perceived competence at the personal layer; overprotective family responses may further reduce agency at the relational layer; and inaccessible services may convert functional limitation into structural exclusion.

Ageism exemplifies this evolving process. Stereotype embodiment theory suggests that age stereotypes are internalized over the lifespan, become self-relevant in later life and impact functioning via psychological, behavioral and physiological routes (Levy, 2009). Studies over time have associated more positive views on aging with increased longevity and improved health outcomes (Levy et al., 2002; Westerhof et al., 2023). In a self-worth systems model, ageism is significant because it provides a cultural narrative that leads older adults to view dependency, slowness, or illness as signs of reduced worth. Self-worth regulation is influenced not only by internal psychological factors but also by cultural interpretations that view later life as either a period of ongoing development, contribution, and connection, or as a time of decline and fading from society. The essence of this model is based on mattering. The sense of being important to others is strongly connected to purpose, depression, and well-being in older adults (Dixon, 2007). It is not the same as getting assistance. Mattering is the feeling that one is important, recognized, and has an impact on others. This difference is crucial in later years since older individuals might get a lot of care but still feel socially unneeded. A care system might ensure a person’s safety without acknowledging the importance of their choices, stories, or contributions. Alternatively, the chance to advise, educate, volunteer, be involved in family choices, or assist peers might uphold self-value by reestablishing mutual exchange and social acknowledgment (Flett and Heisel, 2021).

**Figure 1:**
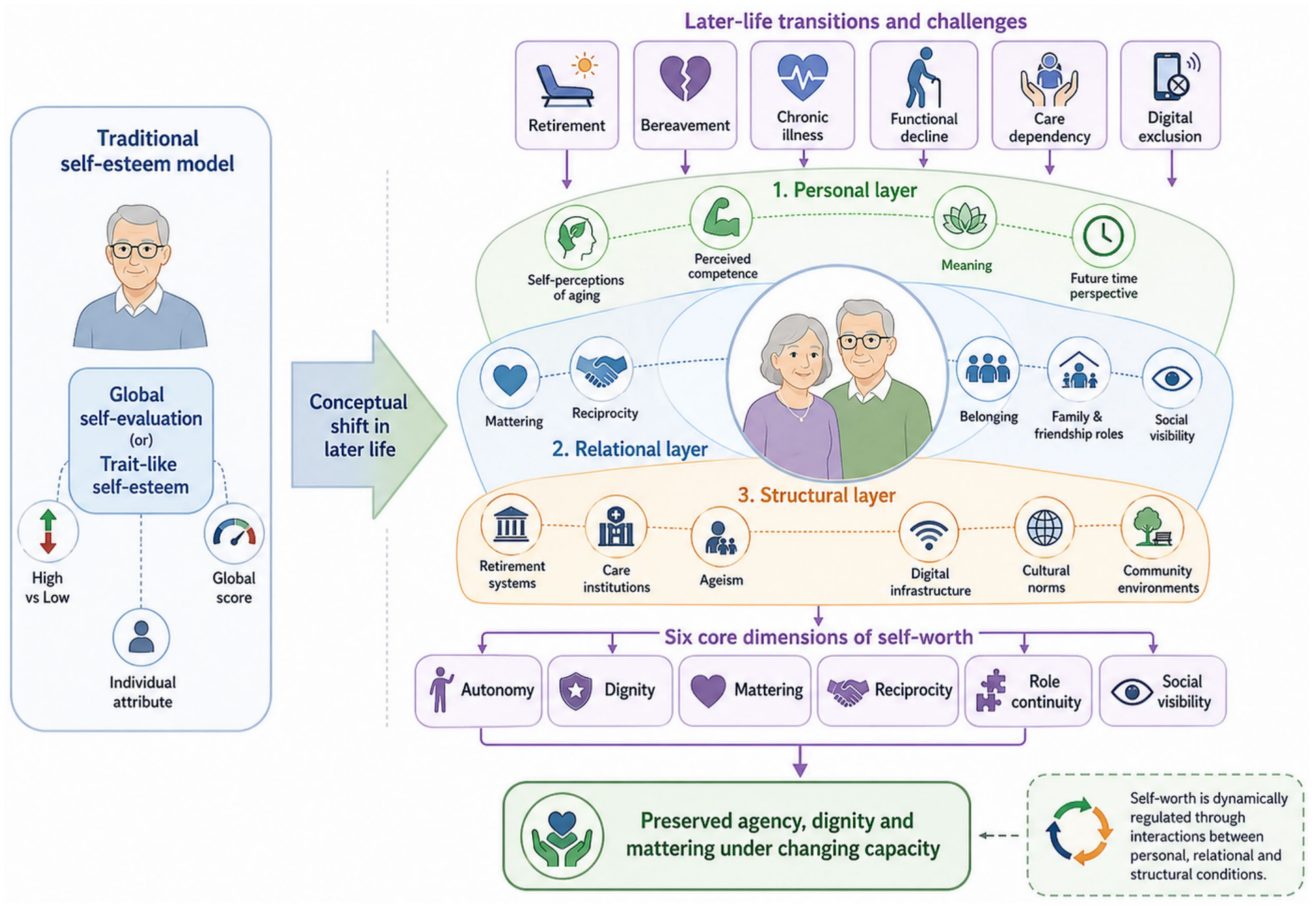
From self-esteem to self-worth systems in later life. The figure contrasts a traditional model of self-esteem as global self-evaluation with a multilevel self-worth systems model. Later-life self-worth is shaped by personal, relational and structural layers and organized around autonomy, dignity, mattering, reciprocity, role continuity and social visibility.

The dynamic model thus views self-worth in later life as a regulatory system that includes both risk and recovery pathways. Risk pathways encompass loss of roles, internalized ageism, becoming socially invisible, one-sided dependency, depersonalization in institutions, and being excluded from decision-making. Pathways to recovery involve care that fosters autonomy, communication that maintains dignity, reciprocal social roles, participation across different generations, environments that are accessible, and contributions that hold cultural significance. The main result is not just increased self-esteem, but maintaining agency, dignity, and significance despite changing abilities (Figure 1).

#### 3.1.4 Relationship to existing frameworks and distinct contribution

The proposed framework builds on, but is not identical to, several established theories. Sociometer theory conceptualizes self-esteem as a monitor of perceived relational value (Leary et al., 1995; Leary, 2005). Self-determination theory identifies autonomy, competence and relatedness as basic psychological needs (Ryan and Deci, 2000). The WHO healthy aging framework emphasizes functional ability as the interaction between intrinsic capacity and enabling environments (Beard et al., 2016). Ecological systems theory situates development within nested environmental systems, while social-ecological models in health promotion emphasize interactions across individual, interpersonal, organizational, community and policy levels (Bronfenbrenner, 1979; McLeroy et al., 1988). Successful aging models have emphasized low disease and disability, high physical and cognitive function, and active engagement with life, but they have also been criticized for potentially privileging independence, productivity and individual responsibility in ways that may marginalize frailty, disability and dependency (Rowe and Kahn, 1997; Martinson and Berridge, 2015; Katz and Calasanti, 2015). These frameworks are essential antecedents rather than competing alternatives.

The contribution of the self-worth systems framework is more specific. It treats later-life self-esteem as one signal of a broader worth-regulation system and specifies the dimensions through which worth is preserved or eroded in aging societies: autonomy, dignity, mattering, reciprocity, role continuity and social visibility. Unlike general ecological frameworks, it focuses specifically on the social production of perceived value (Bronfenbrenner, 1979; McLeroy et al., 1988). Unlike models of successful aging that emphasize low disease burden, high function and active engagement, it explicitly separates dependency from devaluation (Rowe and Kahn, 1997; Katz and Calasanti, 2015). Unlike dignity-centered care models, it extends the analysis beyond clinical encounters to retirement, digital systems, cultural change, ageism and public policy (Chochinov, 2002; Lothian and Philp, 2001; Clancy et al., 2021; Dong et al., 2021). In this sense, the framework does not replace existing theories; rather, it integrates them around a more specific question: how do aging societies communicate to older adults that they remain valued, agentic and consequential? (Table 2).

**Table 2:** Relationship between the proposed framework and existing frameworks.

Existing framework	Main focus	Limitation for the present question	Added value of self-worth systems framework
Sociometer theory	Self-esteem as a signal of relational value.	Less focused on aging transitions, care dependency and institutions.	Treats self-esteem as one signal within a wider later-life worth system.
Self-determination theory	Autonomy, competence and relatedness as basic needs.	Less attention to ageism, long-term care, retirement and digital exclusion.	Embeds psychological needs in later-life institutions and social roles.
WHO healthy aging framework	Functional ability through intrinsic capacity and enabling environments.	Psychosocial value, dignity and mattering are not central outcomes.	Adds perceived worth, dignity and reciprocity as components of healthy longevity.
Ecological/social-ecological models	Multilevel person-environment interactions.	Broad framework not specific to perceived worth in later life.	Specifies worth-relevant mechanisms and feedback loops.
Successful aging models	Low disease/disability, high function and social/productive engagement.	May marginalize frailty, dependency and disability when interpreted normatively.	Separates dependency from devaluation and includes dignity under vulnerability.
Person-centered and dignity-centered care	Recognition of personhood in clinical/care settings.	Often focused on care settings rather than broader aging societies.	Extends dignity logic to community, retirement, digital and policy contexts.

#### 3.1.5 Why “systems”: feedback, non-linearity and path dependence

The term “systems” is used here in a specific sense. Later-life self-worth is not assumed to be the additive result of isolated factors. It is produced through feedback loops among personal interpretations, relational responses and structural arrangements. For example, functional decline may reduce perceived competence; overprotective family responses may further restrict agency; reduced agency may lower self-worth; and lower self-worth may reduce social initiative, thereby deepening isolation. Similarly, digital exclusion may produce embarrassment, avoidance and further skill loss, while ageist assumptions may reduce participation and then be interpreted as evidence that older adults are disengaged. These are circular rather than linear processes.

The framework therefore emphasizes at least five systemic dynamics: loneliness–self-worth feedback, dependency–agency feedback, ageism–internalization feedback, digital exclusion–avoidance feedback and reciprocity–mattering feedback. Negative loops can accelerate withdrawal and perceived devaluation, whereas positive loops can restore participation, contribution and social visibility. This systemic interpretation differentiates the framework from a simple list of psychosocial risk factors.

### 3.2 Social architectures of self-worth in aging societies

#### 3.2.1 Social connection as self-worth calibration

Social connection is commonly portrayed as a safeguard in older age, yet this viewpoint is insufficient for grasping self-worth in societies with aging populations. Social relationships offer emotional support, practical help and companionship. They also evaluate older adults’ visibility, need, respect and importance. In this context, social interaction adjusts one’s sense of self-value. It provides the interpersonal proof that older individuals use to determine if they still have significance in families, communities, care systems, and society at large.

This calibration function is crucial as social networks in later life often undergo changes in density, structure, and meaning. According to the convoy model of social relations, individuals navigate life accompanied by changing networks of intimate and less intimate relationships that vary with personal development and historical context (Antonucci et al., 2014). For seniors, these networks might be altered by factors such as retirement, losing a spouse, children moving away, health issues, disabilities, moving into care facilities, or being left out of digital advancements. These changes are not merely about reducing the amount of social interaction. They might change the level of recognition accessible to the individual. Self-worth can be maintained by a smaller, reciprocal network, whereas a larger, superficial, or condescending network might not achieve this.

The connection between social relationships and health and mortality highlights the importance of viewing social connections as a fundamental component of healthy aging, rather than just a secondary psychosocial factor. Meta-analytic evidence indicates that stronger social relationships are associated with substantially greater survival likelihood (Holt-Lunstad et al., 2010), while sociological and public health syntheses show that social ties influence mental health, health behaviors, physical health and mortality risk across the life course (Umberson and Montez, 2010; Holt-Lunstad, 2022). From a self-worth systems viewpoint, the impact of social relationships on health might be influenced by their ability to maintain agency, reciprocity, and a sense of significance. Social interactions that convey burden, dependency, or low status might not safeguard self-esteem as effectively as interactions that convey respect, usefulness, and a sense of belonging.

This differentiation is vital for societies with aging populations. A model based solely on support may portray older adults as passive care recipients. The self-worth model inquires whether older adults are still given meaningful roles by their social environments. Self-worth infrastructures can be found in family relationships, community groups, religious organizations, neighborhood ties, peer networks, volunteering systems and intergenerational programs when they provide avenues for recognition and contribution. Social connection should be seen not just as being around others, but as having relationships that affirm: “you are still a part of this world, and your presence continues to make a difference.” However, the evidence is stronger for associations between social relationships and mortality or mental health than for direct tests of self-worth as the mediating mechanism. Future studies should therefore distinguish contact frequency, perceived support, mattering, reciprocity and dignity rather than treating social connection as a single exposure.

#### 3.2.2 The loneliness–self-worth loop

Loneliness is often described as the personal discomfort experienced when one’s social connections do not meet their expectations. Within a self-worth systems framework, loneliness is more than just a feeling caused by social isolation; it also endangers one’s perceived relational value. Meta-analytic evidence shows that loneliness, social isolation and living alone are associated with increased mortality risk (Holt-Lunstad et al., 2015), while reviews of later-life loneliness link perceived social isolation to depression, cognitive vulnerability, physiological dysregulation and poorer health outcomes (Ong et al., 2016; Cacioppo and Hawkley, 2009). These results indicate that loneliness is more than just a personal emotion; it is a psychosocial state where one’s perceived social standing becomes uncertain.

The cycle of loneliness and self-worth starts when older individuals get less acknowledgment. Experiencing loss, retiring, falling ill, losing hearing, facing mobility issues, or moving can diminish daily interactions that previously affirmed one’s identity and sense of purpose. With a decrease in social feedback, older adults might start to believe they are less necessary, less engaging, or less important socially. When people feel they matter less, it can decrease their social initiative, making them less likely to connect with others, join community activities, or seek help. Pulling away from social interactions decreases recognition, intensifies loneliness, and further diminishes self-esteem. This loop is not merely about loneliness causing low self-esteem; it’s a recurring process where feeling socially disconnected decreases one’s perceived value, which in turn reduces the motivation and confidence to re-establish connections.

Cognitive and emotional mechanisms strengthen this cycle. Studies on perceived social isolation indicate that loneliness intensifies social threat sensitivity, negative social expectations, and self-protective cognitive biases, which can have the paradoxical effect of diminishing opportunities for reconnection (Hawkley and Cacioppo, 2007). Long-term studies also show future links between loneliness and depression symptoms in middle-aged and older adults (Cacioppo et al., 2010). The evidence indicates that loneliness can be self-sustaining: a person experiencing loneliness might not just have fewer social contacts but could also perceive social situations as likely to result in rejection or being undervalued.

The significance for intervention is crucial. Boosting social interactions might not be enough if the intervention fails to enhance self-esteem. Evidence from meta-analyses indicates that interventions targeting maladaptive social cognition can be particularly successful in alleviating loneliness (Masi et al., 2011). From the viewpoint of self-worth systems, effective interventions should focus on creating environments where older adults feel seen, heard, needed, and respected, rather than just increasing their social interactions. A community lunch may temporarily reduce isolation, but programs that involve older adults in teaching, mentoring, organizing, advising or contributing may more directly disrupt the loneliness–self-worth cycle. This remains a testable hypothesis rather than an established causal conclusion, because many intervention studies measure loneliness or well-being but do not directly measure mattering, dignity or perceived social value.

#### 3.2.3 Reciprocity, usefulness and the need to matter

A key flaw in numerous aging models is their focus on the support received while neglecting the support provided. Seniors are commonly portrayed as care recipients, but their sense of self-worth is significantly tied to reciprocal relationships. To matter means not just being cared for, but also feeling that your actions, thoughts, memories, work, love, or presence impact others. Mattering research has shown that older adults’ perceived mattering is closely related to purpose in life, depression and wellness (Dixon, 2007), while later work has argued that feeling valued rather than expendable is fundamental to older adults’ mental health and well-being (Flett and Heisel, 2021).

The concept of usefulness is a significant aspect of feeling important. Longitudinal studies suggest that feelings of usefulness to others predict later disability and mortality risk among older adults (Gruenewald et al., 2007), and evidence from China shows that self-perceived uselessness is associated with higher mortality risk in older populations (Gu et al., 2017). These results should not be seen as straightforward proof that older individuals need to stay traditionally ‘productive’ to be considered valuable. They imply that societies ought to create diverse, age-inclusive methods for older adults to make contributions. Usefulness can manifest in various forms such as grandparenting, offering emotional support, engaging in community activities, storytelling, mentoring, volunteering, assisting neighbors, cultural transmission, or civic involvement.

It’s important to differentiate between giving and receiving. Future evidence indicates that offering social support might be more closely linked to a longer life than just receiving it (Brown et al., 2003). Likewise, active social participation and volunteering are associated with improved well-being and health outcomes in the elderly (Glass et al., 1999; Anderson et al., 2014). In programs such as Experience Corps, older volunteers are viewed as community assets, where their time, experience and social visibility bring benefits to younger generations while improving their own health and engagement (Fried et al., 2004).

Thus, a self-worth systems model changes the intervention aim from ‘supporting older adults’ to ‘creating reciprocal environments.’ In these environments, seniors get assistance when necessary, but they also have chances to offer help, make decisions, provide advice, and contribute. This is especially crucial in long-term care and family caregiving settings, where support, though well-meaning, can become psychologically damaging if it eliminates all chances for personal agency. The danger lies not in dependency itself, but in one-sided dependency: a situation where the elderly receive care but lose the capacity to impact others significantly. To safeguard self-esteem, it’s essential to create relationships and institutions that maintain mutual presence, even when vulnerability is present.

However, evidence on usefulness, volunteering and giving support is heterogeneous in design and may be confounded by baseline health, socioeconomic status and social resources. Future studies should test whether perceived mattering mediates these associations rather than assuming that all forms of contribution protect self-worth equally.

#### 3.2.4 Retirement and the reconstruction of identity

Retirement significantly impacts self-esteem in later life as it alters the institutional basis of one’s identity. Employment offers benefits beyond just earning money. It schedules time, confirms competence, assigns people to social networks, distributes responsibilities, and provides a socially accepted answer to “who am I?” Psychological research points out that retirement is a process of decision-making, transition, adjustment and reconstruction of roles (Wang and Shi, 2014). For certain people, retirement brings a sense of liberation, relief, and a new sense of purpose, while for others, it signifies a loss of status, reduced social interactions, and a fading from important roles.

The impact of retirement on self-esteem is partly influenced by how much one’s identity is tied to job performance. If work has been the main source of acknowledgment, retirement might eliminate not just duties but also the audience, routines, and social validation of skills. Research on role identity processes indicates that retirees undergo identity work as they transition from roles centered around work to new or modified non-work roles (Bordia et al., 2020). From this perspective, adjusting to retirement involves more than just emotionally adapting to free time; it requires rebuilding a self that stays consistent despite losing a significant social role.

Involvement in social activities seems crucial to this rebuilding. Meta-analyses on retirement adjustment highlight social involvement, marital status, physical well-being, financial situation, and conditions of exit as key factors influencing adjustment (La Rue et al., 2022). Research over time shows that keeping social connections after retiring is related to a decreased chance of mortality (Steffens et al., 2016). These results align with a self-worth systems perspective: retirement is less harmful when older individuals maintain or create new social identities that offer a sense of belonging, competence, and acknowledgment. Participation in community organizations, volunteer work, part-time employment, caregiving, religious involvement, educational pursuits, and creative activities can each function as identity connections between the working world and life beyond work.

New research challenges the notion that retirement causes an existential decline. Retirement may provide new purpose to people, particularly if they can reorganise time and activity around meaningful goals, a longitudinal study suggests (Yemiscigil et al., 2021). Thus, the key problem is not retirement itself, but how it is socially framed as either exiting a role or transforming it. Viewing retirement as a retreat from being useful might harm one’s sense of self-worth, whereas seeing it as a shift to new ways of contributing can preserve or boost self-worth.

This distinction holds policy importance for aging populations. While financial planning for retirement is essential, it is not enough. There is a need for societies to engage in social and identity planning, which supports older individuals in maintaining their roles, group memberships, routines, contributions, and visibility post-retirement. Preparing for retirement should encompass not just pensions and healthcare, but also chances for involvement after retirement, contributions across generations, continuous learning, and community participation. By adopting a self-worth systems approach, retirement is reimagined not as the end of being productive but as a vital opportunity to rebuild identity, ensure relevance, and reimagine the social value of aging.

The evidence also cautions against treating retirement as uniformly harmful or beneficial. Its implications for self-worth likely depend on voluntariness, financial security, health status, prior work identity and availability of alternative roles.

### 3.3 Threats to self-worth across later life

#### 3.3.1 Ageism and internalized devaluation

Ageism poses a significant threat to self-esteem in older adults as it turns the natural and social aspects of aging into a culturally undervalued identity. It functions not only through clear discrimination but also through daily assumptions that older individuals are less competent, adaptable, productive, technologically skilled, or deserving of investment. From a self-worth systems viewpoint, ageism is not just a bias; it functions as a societal mechanism that determines whose agency, contributions, and future opportunities are acknowledged.

##### 3.3.1.1 From stereotype exposure to self-worth threat

Understanding the internalization of ageism is facilitated by the central mechanism of stereotype embodiment theory. According to this view, age-related stereotypes are internalized over a lifetime, may function without conscious awareness, gain personal relevance in older age, and impact functioning through mental, behavioral, and physical channels (Levy, 2009). This implies that negative cultural messages about aging are not just external affronts. As time passes, these factors might integrate into the way older adults perceive their own slowness, reliance on others, health issues, or social isolation.

There is now strong evidence supporting the health implications of ageism. A comprehensive review of studies from various countries discovered that ageism was linked to notably poorer health outcomes in most of the associations analyzed (Chang et al., 2020). Ageism has been identified as a social determinant of health, with its institutional, interpersonal, and self-directed forms interacting within social systems (Mikton et al., 2021). From a self-worth perspective, this is important since ageism not only predicts declining health but also offers a societal narrative that may cause older individuals to perceive themselves as burdens, outdated, or less entitled to autonomy.

##### 3.3.1.2 Internalized devaluation and diminished agency

Internalized ageism is especially harmful when it turns age-related changes into self-deprecation. Experiencing functional slowing, memory issues, chronic illness, or reduced mobility might not just be seen as challenges, but as proof that one is ‘less of a person.’ Viewing aging positively has been associated with longer survival and better recovery from disabilities, indicating that older adults’ perceptions of aging can affect how they handle challenges in later life (Levy et al., 2002; Levy et al., 2012). In contrast, negative perceptions of aging might diminish motivation, decrease health participation, and restrict future possibilities.

Ageism is conceptualized as a danger to agency in the self-worth systems model. It limits the actions that older adults view as socially acceptable. Elderly individuals might steer clear of physical activity, online education, engaging in social activities, exploring sexuality, pursuing learning, volunteering, or being publicly visible because these pursuits seem to clash with age-related stereotypes. Hence, ageism not only communicates to older adults how they are viewed by others but also restricts their sense of entitlement to pursue certain actions. A further caution is that the evidence base combines experimental stereotype-activation studies, longitudinal studies of self-perceptions of aging and observational studies of ageism. These designs do not support identical causal claims. In this review, ageism is therefore treated as a plausible self-worth threat operating through cultural meaning, interpersonal treatment and internalized expectations, rather than as a single uniform exposure.

#### 3.3.2 Functional decline, dependency and dignity threat

Functional decline is frequently seen as a direct cause of diminished well-being, yet its effect on self-esteem largely hinges on the social interpretation and management of the decline. Having physical impairments, frailty, or cognitive changes does not inherently strip away dignity. The more significant danger occurs when deterioration is paired with a loss of autonomy, being treated like a child, breaches of privacy, becoming socially invisible, or being excluded from decision-making.

##### 3.3.2.1 Dependency without devaluation

A significant mistake in aging research is equating autonomy with independence. Numerous seniors need support with mobility, personal care, medication management, transportation, financial matters, or digital technology. Needing help does not automatically equate to a loss of self-determination. The moral dilemma is maintaining independence while being interconnected. Classical discussions of older people’s dignity and autonomy in healthcare already emphasized that dignity can be undermined when older patients are treated as passive, voiceless or inconvenient (Lothian and Philp, 2001). Subsequent research on dignity among older adults indicates that it is relational, embodied, and context-sensitive, rather than merely a personal attitude (Clancy et al., 2021).

The crucial difference is thus between dependency and devaluation. Dependency is the requirement for assistance, while devaluation happens when this requirement is seen as a diminishment of one’s identity. Within a self-worth systems framework, dependency in later life becomes damaging to one’s psyche when it implies that the elderly individual is no longer a decision-maker, contributor, or moral equal.

##### 3.3.2.2 Relational autonomy and supported agency

In this situation, the debate between autonomy-centered and relational autonomy frameworks becomes central. Pullman contended that autonomy and dignity in long-term care cannot be fully grasped through an individualistic perspective, as care dependency establishes relational and institutional contexts that influence decision-making (Pullman, 1999). Sherwin and Winsby expanded on a relational perspective of autonomy for nursing home residents, highlighting that social relationships, institutional routines, and power dynamics enable or hinder autonomy (Sherwin and Winsby, 2011). This debate matters because an autonomy-centered framework may overvalue independent choice, whereas a purely protective care framework may justify paternalism. A relational autonomy perspective offers a middle position: older adults may require assistance, but their preferences, histories, relationships and decision-making authority should still shape care (Mackenzie and Stoljar, 2000). The self-worth systems framework adopts this relational position and treats supported agency, rather than independence alone, as the relevant condition for dignity.

This viewpoint bolsters the self-worth argument by suggesting that autonomy is not merely an internal capacity for decision-making in older individuals; it is a condition upheld by social support. Seniors may preserve their self-esteem when they are asked about their preferences, engaged in care planning, provided with meaningful options, and treated as individuals with life stories. Their self-esteem may diminish if aid is given in ways that violate their privacy, overlook their preferences, or turn them into mere subjects of control.

#### 3.3.3 Long-term care and institutional erosion of self-worth

In long-term care facilities, various factors threaten self-respect, including dependency, regimented schedules, surveillance, bodily exposure, lack of privacy, separation from one’s home, and unequal power dynamics between residents and staff. While these settings can safeguard life and safety, they might undermine identity if care prioritizes efficiency over individuality.

##### 3.3.3.1 From personhood to task management

Institutional care may inadvertently reduce older adults to mere residents, beds, diagnoses, or care tasks, stripping them of their personal histories. This change is significant because self-esteem relies on being acknowledged as a unique individual, not just as a body needing upkeep. A comprehensive review of autonomy in residential care revealed that perceived autonomy includes the ability to make daily life choices, influenced by personal abilities and the support from family and care settings (Moilanen et al., 2021). Research in long-term care settings indicates that dignity is linked to physical, psychological, autonomous, social, caring, and value-related aspects (Dong et al., 2021).

So a series of small, repeated losses can corrode institutional self-worth: being dressed without consultation, spoken about rather than spoken to, having no control over meal times, being stopped from managing small risks, being seen as incapable before capacity is established. While these actions may seem trivial from an administrative perspective, they collectively indicate that personal preferences are being ignored.

##### 3.3.3.2 Safety, risk and overprotection

A fundamental challenge in long-term care is maintaining a balance between safety and independence. Preventing risks is important, but too much protection can result in losing one’s dignity. If routines for fall prevention, medication, or institutional schedules remove all meaningful decisions, care could sustain biological life while restricting personal agency. Evidence from residential and long-term care settings indicates that dignity and autonomy are associated with daily choice, involvement in decision-making, respectful relationships, privacy and the possibility of accepting dependency without losing personhood (Dong et al., 2021; Moilanen et al., 2021).

This differentiation is particularly crucial in dementia care. Ensuring dignity in dementia care involves safeguarding individuals from harm while acknowledging their remaining autonomy, personal preferences, and relational identity (Heggestad et al., 2015). According to a self-worth systems perspective, quality long-term care should not be evaluated solely based on safety, cleanliness, or medical results. It should also be assessed based on whether residents continue to be socially visible, relevant in decision-making, and regarded as individuals whose histories and preferences still guide their care.

#### 3.3.4 Digital exclusion and technological humiliation

The rise of digitalization has formed a new framework for threats to self-esteem. Digital skills are becoming essential for health appointments, banking, transportation, social interactions, public services, shopping, entertainment, and communication. For seniors who are left out of these systems, the issue extends beyond just access. Being digitally excluded can often convey reliance, ineptitude, and becoming socially outdated.

##### 3.3.4.1 Digital exclusion as loss of agency

Lacking digital access can frequently imply dependence, incompetence, and social obsolescence (Ge et al., 2025). It may limit access to medical services, social involvement, and daily independence. In terms of self-esteem, digital exclusion is harmful as it turns everyday tasks into frequent instances of failure or dependence on others. An elderly individual who struggles with online registration, mobile payments, telehealth, transportation apps, or authentication systems might face not just inconvenience but also embarrassment.

Research indicates that perceived ageism is linked to internet usage among the elderly, suggesting that digital exclusion is influenced by age-related stereotypes rather than just age or lack of skills (Choi et al., 2020). If seniors are consistently told, whether explicitly or implicitly, that digital platforms are ‘not suitable for them,’ they may come to view their lack of use as a reflection of personal shortcomings. This is why digital exclusion needs to be seen as an issue that is both technological and psychosocial.

##### 3.3.4.2 Digital ageism and design-based devaluation

Digital ageism is the age-related prejudice found in technology design, data, language, and application. A scoping review on digital technology design revealed a gap between the ideal of involving older adults and the reality of design practices, with ageist assumptions affecting technology development and the inclusion of older users (Mannheim et al., 2023). Digital ageism in AI may develop due to data that lacks representation, design choices that exclude, and the belief that older adults are not key stakeholders (Chu et al., 2022).

The idea of technological humiliation aids in understanding the experiential aspect of this process. This pertains to the humiliation, irritation, or diminished dignity that older people feel when they must interact with systems created for younger, faster, and more digitally adept users. The problem is not that seniors cannot learn technology; it’s that many digital systems are created as if they do not include older adults. Within a self-worth systems framework, digital inclusion goes beyond just being a skills intervention; it is an intervention for dignity.

#### 3.3.5 Cultural disruption in east Asian and Chinese interdependent aging contexts

Cultural influences organize how self-worth is recognized in later life. In this section, the discussion focuses primarily on East Asian and Chinese interdependent contexts, where filial norms, family role continuity and intergenerational obligation have historically shaped older adults’ perceived value. The argument should not be generalized to all interdependent societies. Rather, East Asian examples are used to show how cultural recognition systems can preserve self-worth through respect and family centrality, but can also generate pressure when older adults feel that they have become a burden. Traditional sources of later-life value are being reshaped by modernization, migration, urbanization, smaller family size, women’s labor-force participation and digital transformation.

##### 3.3.5.1 Filial piety as a recognition system

Filial piety has historically acted as both a caregiving norm and a recognition framework in many East Asian and Chinese cultural contexts. It informs older adults that they continue to hold moral value, remain central to the family, and have significance across generations. Modern psychological frameworks differentiate between reciprocal and authoritarian types of filial piety, indicating that filial relationships can foster care and respect, but may also create tension when duty, hierarchy, and independence clash (Bedford and Yeh, 2019).

Research indicates that filial piety still impacts the well-being and loneliness of older adults, though its significance is evolving in different cultural and social settings (Ren et al., 2022). For instance, older Chinese immigrants might hold onto filial expectations but also adjust or redefine these expectations due to migration, generational gaps, and evolving family dynamics (Zhang, 2022). In the context of Chinese seniors, perceived filial piety involves care, respect, greetings, happiness, obedience, and financial aid, with respect often standing out (Dong et al., 2014). This reinforces the idea that in interdependent cultures, self-worth is derived not just from material support, but also from respect and symbolic acknowledgment.

##### 3.3.5.2 Autonomy, interdependence and the burden script

Cultural disruption arises when older individuals find themselves torn between traditional family respect and modern demands for independence, efficiency, and avoiding being a burden. In communities that depend on mutual support, family care can uphold self-esteem when viewed as part of ongoing reciprocity. Yet, such care can negatively impact self-esteem if elderly individuals feel they are a financial strain on their children, disrupting family development or failing to contribute.

This situation imposes a dilemma where the elderly are culturally entitled to receive care but may feel a moral obligation to avoid needing it. Demographic shifts, smaller family sizes, geographic mobility, and changes in women’s workforce participation exacerbate this tension. The outcome is not merely a reduction in filial piety, but rather a restructuring of mutual dependence. While older adults may still cherish family ties, their sense of self-worth is more reliant on experiencing interdependence as mutual acknowledgment rather than a begrudging duty.

##### 3.3.5.3 Modernization and role displacement

Research from various cultures indicates that societies have different interpretations of aging and well-being. Research comparing Japan with the United States shows that cultural settings affect how seniors perceive growth, purpose, and social relationships as they age (Karasawa et al., 2011). Traditional status systems may be weakened by modernization, but not entirely replaced by new roles. When seniors simultaneously lose their work identity, household control, tech skills, and central family role, their self-esteem is at risk due to role displacement.

**Figure 2:**
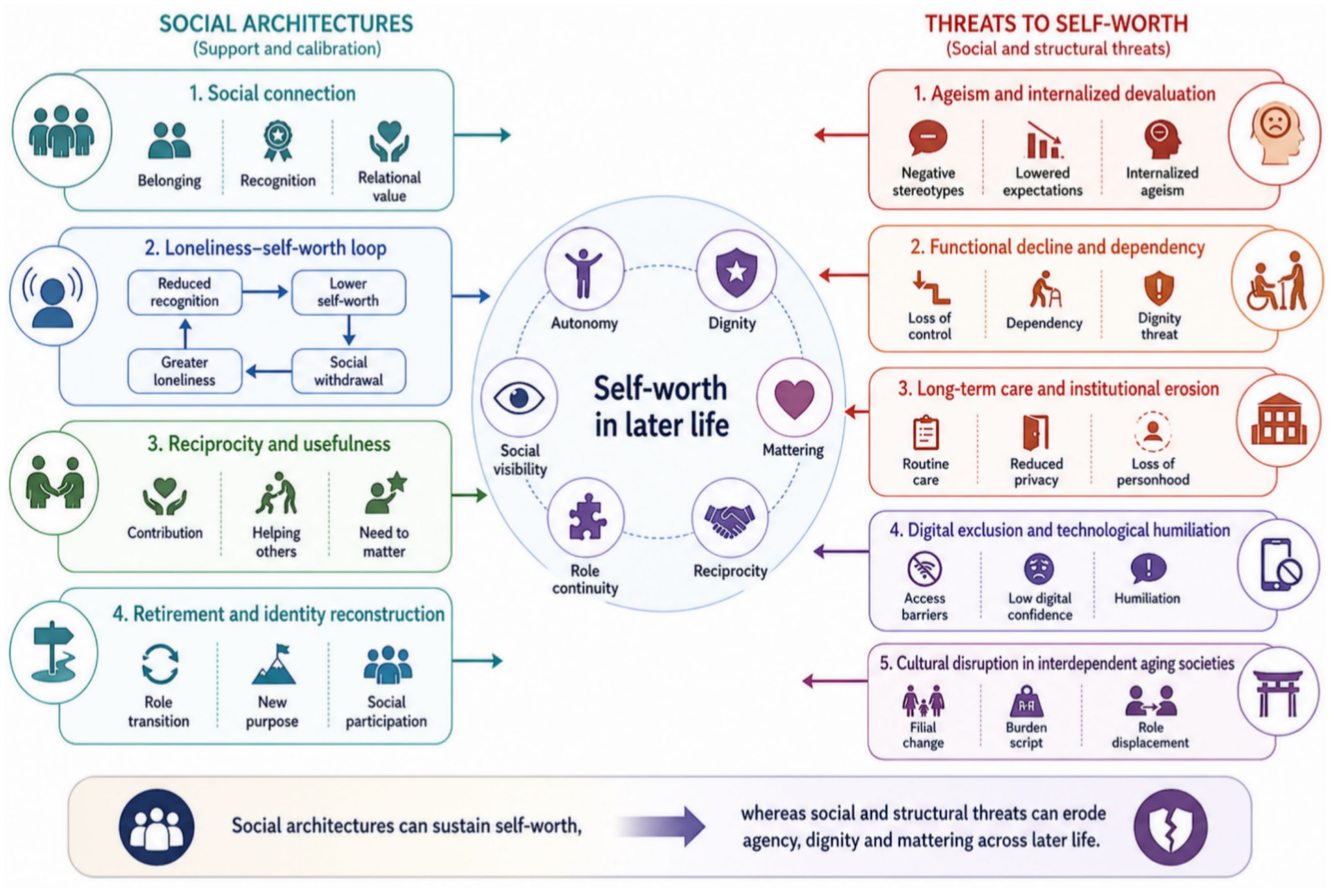
Social architectures and threats to self-worth in aging societies. The figure summarizes social processes that may support self-worth, including reciprocity, participation and identity reconstruction, and threats that may undermine it, including loneliness, ageism, dependency, institutional erosion, digital exclusion and cultural disruption.

This suggests that research on self-worth that is culturally sensitive should steer clear of two oversimplifications. Firstly, it is important not to idealize traditional cultures as consistently protective, because filial systems can also impose pressure, hierarchy, and gendered roles. Secondly, it should not be presumed that Western models centered on autonomy reflect the concept of self-worth in societies that are interdependent. For numerous seniors, the aim is not to be independent from others, but to have meaningful involvement in relationships. A comprehensive analysis of self-worth in aging societies should explore how cultural norms of autonomy, interdependence, respect, and usefulness are being reinterpreted in the face of demographic and technological changes (Figure 2).

### 3.4 Pathways from self-worth to health and healthy longevity

Self-worth should not be treated as either a simple health outcome or a proven causal determinant of longevity. Rather, it is best understood as a regulatory construct that may influence how older adults interpret and respond to illness, dependency, social loss and functional change. The evidence reviewed below is uneven. Psychological and social pathways are supported by relatively stronger longitudinal and meta-analytic evidence from adjacent constructs such as self-esteem, loneliness, social connection and purpose. Behavioral and cognitive pathways are plausible but often indirect. Biological embedding is the most speculative pathway and should be interpreted cautiously. The framework therefore proposes self-worth as a potential integrative mechanism linking social experience with healthy aging, not as a single independent cause of longevity.

#### 3.4.1 Psychological pathways: depression, anxiety and resilience

The journey from self-esteem to health starts with managing emotions. Research consistently shows that low self-esteem is a precursor to depression and anxiety, with longitudinal meta-analyses favoring the vulnerability model over the reverse scar model (Sowislo and Orth, 2013). Self-esteem across one’s lifespan is a predictor of significant outcomes like mood, depression, relationship satisfaction, and physical health (Orth et al., 2012). A central controversy is whether low self-esteem functions primarily as a vulnerability factor for later depression and anxiety or whether psychological distress leaves a “scar” that subsequently lowers self-esteem. Longitudinal meta-analytic evidence has generally supported the vulnerability model more strongly than the scar model, but reciprocal processes remain possible and may vary by age, context and measurement interval (Sherwin and Winsby, 2011). For the present framework, this debate means that self-worth should not be treated simplistically as either cause or consequence. In later life, diminished worth may increase vulnerability to depression, while depression, illness and social withdrawal may also further erode perceived worth. As people age, these relationships can become crucial since threats to self-esteem often coincide with experiences like bereavement, retirement, chronic health issues, functional impairments, and decreased social recognition. A model of self-worth systems expands on this evidence by exploring why self-evaluative vulnerability becomes clinically significant in older adults. Depression in older adults who feel useless, invisible, or burdensome might not just be an internal mood but could also signify their sense of social devaluation. Older adults might also feel anxious when they lose control over their health, finances, mobility, digital systems, or care arrangements. In this situation, self-esteem influences whether losses in later life are seen as manageable changes or as signs of personal fading.

##### 3.4.1.1 Self-worth threat and depressive vulnerability

Later-life depression often stems from a buildup of disruptions in personal agency and social belonging. A limited self-esteem framework would consider low self-esteem as just one of several risk factors. A self-worth systems model views it as an indication that the individual’s relational and role-based foundations have diminished. For instance, an elderly person might experience depression not just due to decreased mobility, but because this loss has resulted in less participation, fewer social invitations, reduced family involvement, and a sense of social irrelevance. Therefore, the pathogenic mechanism involves not just a loss of function, but a loss of function perceived as reduced value. This viewpoint is crucial as it alters the logic of intervention. If low self-esteem is sustained by losing roles, age discrimination, isolation, or one-sided dependency, then cognitive strategies alone might not be enough to enhance self-assessment. Restoring agency, significance, and social recognition might be necessary for psychological recovery. The goal of therapy shifts from just ‘I feel better about myself’ to ‘I have reasons, roles, and relationships that give my life significance.’

##### 3.4.1.2 Resilience as preserved self-worth under adversity

Resilience acts as a positive counterbalance to threats against self-worth. Meta-analytic evidence indicates that trait resilience is associated with better mental health in older adults (Färber and Rosendahl, 2020), and recent systematic review evidence links resilience with successful aging (Trică et al., 2024). According to a large cohort study from the Health and Retirement Study, increased psychological resilience is connected to a lower risk of mortality from all causes in older adults, but the study’s observational design limits causal interpretation (Zhang et al., 2024). Under the existing framework, resilience goes beyond merely an individual’s capacity to rebound. It involves maintaining self-esteem during times of loss, reliance, or unpredictability. Seniors tend to stay resilient when they can view functional changes without devaluing themselves overall, keep meaningful social roles, and receive support without feeling humiliated. Self-worth serves as a mental shield, enabling individuals to view adversity as a challenge to overcome rather than evidence of a diminished life value.

#### 3.4.2 Behavioral pathways: activity, adherence and self-care

Self-esteem influences health through behavior. Health behavior among older adults is commonly viewed as a matter of knowledge, discipline, or access, yet it is also determined by whether people perceive their future as valuable enough to invest in. Seniors who see themselves as important, necessary, and competent might be more motivated to stay physically active, follow treatment plans, attend preventive appointments, and keep up with self-care habits. Alternatively, a decline in self-worth can lead to a reduction in the motivation for self-care. Findings about having a purpose in life connect self-value with behavior. Older adults with a greater sense of purpose are more likely to engage in healthy behaviors, such as physical activity and preventive health measures, and are less prone to unhealthy habits (Kim et al., 2020). Purpose has also been associated with accelerometer-measured physical activity among older adults (Sutin et al., 2025) and with use of preventive health care services (Kim et al., 2014). While purpose and self-worth are not the same, both concepts reflect the motivational feeling that one’s life is meaningful and focused on the future.

##### 3.4.2.1 Self-care as an expression of perceived worth

A self-worth systems model views self-care as encompassing more than just managing diseases. Using medication, going to rehab, maintaining a healthy diet, staying active, keeping track of symptoms, and asking for assistance all suggest a belief in a valuable future. When elderly individuals embrace ageist or burden-related stories, this belief might become less strong. They might postpone seeking care, skip rehabilitation, become inactive, or avoid preventive services because they no longer see themselves as worthy of investment. A recent study focusing on self-worth and health-promoting behaviors in older adults living in the community discovered a statistically significant yet modest link, with socioeconomic factors being stronger predictors (Zarghami et al., 2025). This distinction is significant. While self-worth might affect health behavior, it is not separate from income, education, access, health literacy, and environmental barriers. Hence, self-worth ought to be understood as a motivational path within a wider structure of self-care.

##### 3.4.2.2 Adherence, agency and chronic disease management

Self-worth systems are also related to medication adherence and chronic disease self-management. A systematic review shows a positive link between self-reported health literacy and medication adherence in older adults (Schönfeld et al., 2021). Health literacy goes beyond just technical knowledge; it involves confidence, self-efficacy, and the individual’s perceived capability to navigate medical systems. Elderly individuals who perceive themselves as disregarded, humiliated, or lacking competence may be less motivated to inquire, seek clarification, or adhere to intricate procedures. Engagement serves as the behavioral link between self-worth and a healthy, long life. Self-worth encourages the motivation to take action, observe, stick to plans, seek help, and continue. Such erosion increases the risk of behavioral withdrawal. Poor adherence does not equate to older adults being unmotivated or irresponsible. Health systems must protect agency and dignity to uphold self-care.

#### 3.4.3 Social pathways: participation, belonging and visibility

The social route is crucial as self-esteem is adjusted through relationships. Engaging in social activities gives older adults more than just company; it shows that they are acknowledged, valued, and have an impact. Engaging in social activities is considered a key factor in successful aging and is linked to various aspects of health (Douglas et al., 2017). Challenges to social engagement for seniors include physical restrictions, transportation difficulties, social anxiety, health conditions, environmental limitations, and a lack of inclusive options (Townsend et al., 2021). These obstacles are not just logistical; they also serve as routes that can limit self-esteem.

##### 3.4.3.1 Participation as social visibility

Engagement helps maintain self-esteem by keeping older adults socially active. Visibility involves being in environments where one’s preferences, narratives, abilities, and contributions are recognized. Community groups, volunteering, religious participation, physical activity groups, lifelong learning, cultural activities, and intergenerational programs all treat older adults as people with a role. Research indicates that social relationships affect mental health via mechanisms like belonging, social support, social influence, identity, and meaning (Andersen et al., 2021). A self-worth model combines these mechanisms by highlighting that social involvement is beneficial to health when it reinforces a sense of significance. A group offering identity and recognition might offer more protection than an environment that just boosts contact frequency.

##### 3.4.3.2 Belonging, group identity and health

Research on social identity underscores the significance of belonging to a group. Being part of social groups has been demonstrated to guard against future depression, ease depressive symptoms, and lower the risk of relapse (Cruwys et al., 2013). Retirees who continue their social group memberships after retiring tend to experience a higher quality of life and a lower chance of mortality (Steffens et al., 2016). These results imply that a sense of belonging is not merely a surface-level psychological phenomenon. It gives a system for individuals to grasp their identity, their role, and their significance. As people age, the importance of group identity may increase due to potential changes or reductions in occupational, parental, or caregiving roles. Social groups can act as substitute or complementary identity systems. They assist older adults in preserving a connection between their past and present, while also generating new ways to be seen. The important health pathway is not just social interaction leading to an improved mood. The significant health pathway is not merely social contact resulting in a better mood.

#### 3.4.4 Cognitive pathways: agency, meaning and engagement

Self-worth might indirectly affect cognitive health through aspects like agency, meaning, and engagement. Cognitive aging is influenced by both neuropathology and the way individuals utilize their cognitive abilities in everyday activities. Elderly individuals who sense a sense of purpose and social importance are more likely to seek out stimulation, keep up routines, learn new skills, engage in conversations, solve problems, and remain active in complex settings. A sense of purpose in life is connected to a decreased likelihood of Alzheimer’s disease and mild cognitive impairment in older adults residing in the community (Boyle et al., 2010). The Health and Retirement Study’s longitudinal data indicates that having a sense of purpose in life helps shield older adults from cognitive decline (Kim et al., 2019). A recent systematic review and meta-analysis suggest that social connections influence cognitive patterns in older age (Piolatto et al., 2022). These results do not confirm that self-worth directly averts cognitive decline, but they do support the idea that cognitive engagement might connect meaningful social interactions with healthier cognitive aging.

These findings support a plausible link between meaning, engagement and cognition, but they do not establish that self-worth independently protects against cognitive decline. Reverse causality and shared causes, including education, health status and social resources, remain important concerns.

##### 3.4.4.1 Agency and cognitive effort

This pathway revolves around agency. Older adults are more motivated to plan, remember, communicate, and take action when they believe their decisions are important. In contrast, settings that take away decision-making could decrease opportunities for mental effort. Excessive care, rigid institutional practices, and lack of digital access can diminish the necessity or chance to engage in cognitive activities. Cognitive life could become more restricted, even if essential care requirements are fulfilled. Meaning is equally crucial. Analyses and theoretical summaries on purpose and cognitive aging indicate that having a purpose might aid cognition by structuring goals, encouraging participation, and maintaining behavioral activity (Sutin et al., 2021). From a self-worth perspective, meaningful involvement is not merely a mental task; it is thought connected to importance. Cognitive activity in older adults is more likely when activities are linked to their identity, usefulness, and sense of belonging.

##### 3.4.4.2 Engagement versus passive safety

This difference has practical consequences for care. By concentrating exclusively on safety, systems could unintentionally decrease cognitive engagement by reducing choices, risks, and responsibilities. A self-worth-preserving environment must therefore provide cognitively meaningful participation: choosing routines, contributing to household or institutional life, mentoring, storytelling, learning, creative work, problem-solving and intergenerational exchange. Promoting cognitive health should go beyond puzzles or memory exercises and include chances to stay active in a meaningful social environment.

#### 3.4.5 Biological embedding of self-worth threat

The biological pathway is the most tentative part of the framework and should be interpreted as a set of hypotheses rather than established evidence. Existing studies link self-esteem, loneliness, purpose, social relationships and stress-related biological markers, but they rarely test later-life self-worth as a distinct mechanism. Therefore, biological embedding should be understood as an indirect possibility: repeated experiences of humiliation, exclusion, loneliness or loss of agency may contribute to stress-related, inflammatory, sleep-related or behavioral processes, but current evidence does not establish that self-worth itself is the operative biological cause.

##### 3.4.5.1 Stress physiology and cortisol regulation

There is limited but insightful evidence connecting changes in self-esteem to stress biology in older adults. A long-term study discovered that decreases in self-esteem were linked to elevated daily cortisol levels during psychological stress, while boosts in self-esteem seemed to help regulate cortisol in stressful situations (Liu et al., 2014). This suggests that self-assessment resources might affect the physiological reactions of older adults to stress. In a broader sense, research in stress biology indicates that when stress systems are repeatedly activated, it can be harmful if the responses are chronic, unregulated, or not well resolved (McEwen, 1998). Within a self-worth systems framework, chronic devaluation can be conceptualized as a possible social stressor. However, this remains an inference from broader stress literature rather than direct evidence that self-worth independently alters cortisol regulation. More precise studies are needed to test whether perceived worth predicts stress physiology after accounting for depression, loneliness, socioeconomic disadvantage, illness burden and general perceived stress.

##### 3.4.5.2 Inflammation, loneliness and social threat

Another possible route is inflammation. Loneliness has been linked to inflammatory markers in older adult samples (Van Bogart et al., 2022), and broader psychoneuroimmunological models connect social threat and stress-related processes to inflammation and depression (Slavich and Irwin, 2014). These results are important as threats to self-worth are frequently influenced by social factors. An elderly individual who feels unneeded or like a burden might not only feel sadness but also face ongoing social threats. The intersection of purpose and meaning with inflammatory aging is also possible. Research indicates that shifts in life purpose might be linked to low-level chronic inflammation in older adults (Giannis et al., 2024). This does not mean that having a purpose or self-esteem directly influences inflammation. Instead, it implies that motivational and existential conditions might be linked to biological aging through consistent behaviors, stress management, and health habits.

##### 3.4.5.3 Epigenetic aging and cumulative social experience

**Figure 3:**
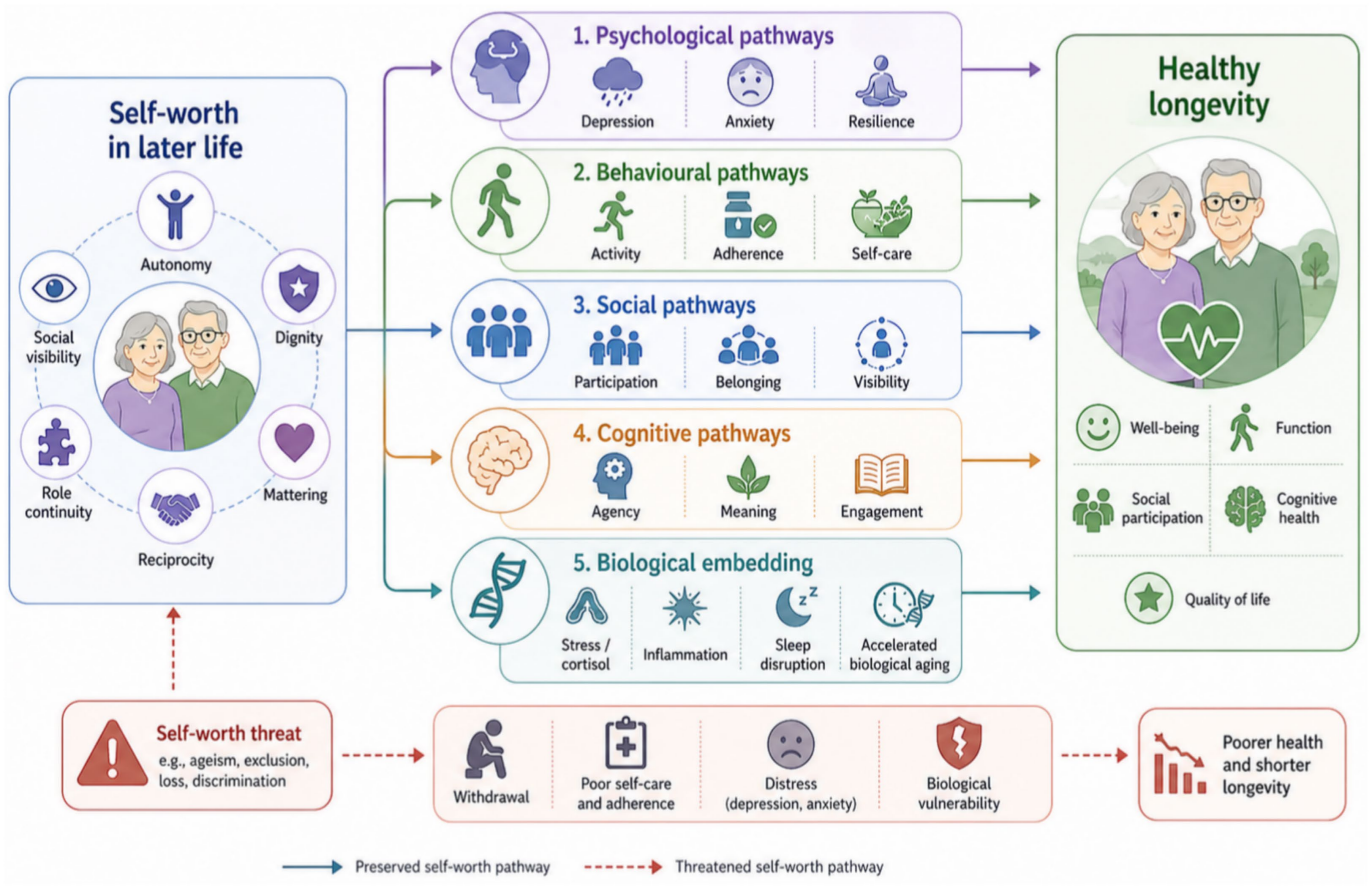
Pathways from self-worth to health and healthy longevity. The figure presents psychological, behavioral, social, cognitive and stress-related biological pathways through which self-worth may be associated with well-being, function, participation, cognitive health and quality of life. Biological pathways are presented as tentative and indirect.

Recent research implies that there could be a connection between social relationships and biological aging markers. According to the Health and Retirement Study, the quality of social relationships is linked to epigenetic aging in older adults (Rentscher et al., 2023). Even though this body of literature is still evolving, it backs a wider assertion: social experiences have psychological effects. Experiences of connection, rejection, recognition, or devaluation that occur repeatedly may build up over time and influence stress biology and aging-related processes. From the perspective of the self-worth systems framework, biological embedding is best treated as a downstream and indirect possibility. Social relationship quality and other psychosocial exposures may be associated with biological aging markers, but the specific contribution of self-worth remains untested. Future research should examine whether perceived worth predicts biological outcomes independently of loneliness, depression, physical illness, socioeconomic status and health behaviors (Figure 3).

### 3.5 Evidence-informed strategies for preserving self-worth in aging societies

#### 3.5.1 Autonomy-supportive environments

Preserving self-worth in aging societies requires more than interventions aimed at increasing individual self-esteem. It requires environments and practices that protect agency, dignity, mattering, reciprocity, role continuity and social visibility. The strategies reviewed below should be understood as evidence-informed rather than definitively proven self-worth interventions. Many studies evaluate outcomes such as loneliness, well-being, quality of life, participation or health behavior, while direct measurement of later-life self-worth remains uncommon. This gap reinforces the need for future trials and implementation studies that explicitly assess self-worth-relevant mechanisms.

It’s important to note this distinction since dependency harms self-worth when it takes the form of passivity. A systematic review of residential care facility autonomy for older adults with physical impairments concluded that autonomy is shaped by personal capabilities and environmental conditions (staff attitudes, institutional routines, physical accessibility, and decision-making opportunities) (van Loon et al., 2021). Therefore, autonomy is not just an internal psychological asset; it is created through interactions, surroundings, and caregiving practices. Even with impairments, an older adult can maintain control if the environment provides options, adaptability, and assistance.

Age-friendly environments apply this concept outside of care facilities. Community planning, transportation infrastructure, accessible living spaces, safe public environments, and participation opportunities can all help preserve self-esteem by keeping older adults active and visible in their daily routines. Findings from Japan, based on mixed-method research, imply that age-friendly settings and community-oriented social innovations can help maintain independence, participation, and dignity in older individuals (Aung et al., 2022). This upholds a wider principle: environments protect self-worth by minimizing the difference between what older adults value and what their surroundings permit them to do.

A self-worth systems approach thus considers autonomy support to be a concern of public health and design. Merely urging older adults to be active or independent is not adequate; societies should dismantle the environmental and institutional barriers that transform typical age-related changes into preventable dependency. Autonomy-supportive settings should ensure mobility is accessible, services are flexible, choices are meaningful, human assistance is available when digital systems fail, and there are opportunities to contribute. According to this model, autonomy does not mean being separate from others, but having the ability to act as an agent within supportive connections.

#### 3.5.2 Dignity-centered care

Dignity-focused care reflects the clinical and institutional commitment to preserving self-respect. Autonomy defends an older person’s ability to act, whereas dignity upholds their status as someone whose life, history, body, preferences, and voice should be respected. In situations where elderly people face physical exposure, dependency, cognitive shifts, routine care, and unequal power dynamics with professionals or family caregivers, this is especially significant.

Dignity-centered practice is grounded in person-centered care. A comprehensive review of literature on person-centered care for elderly individuals with chronic illnesses and functional limitations highlighted personal choice, autonomy, respect, coordinated care, and consideration of individual preferences as key principles (Kogan et al., 2016). Findings from dementia care imply that person-centered care strategies can decrease agitation, neuropsychiatric symptoms, and depression, and simultaneously improve quality of life (Kim and Park, 2017). The importance of these findings lies in showing that acknowledging personhood goes beyond being an ethical ideal and is linked to meaningful clinical results. Dignity-focused care needs to address the subtle practices that can either uphold or undermine a person’s self-worth. Patient-reported dignity during hospital admissions is facilitated by key factors such as communication, privacy, respect, involvement in decision-making, and relational care, according to quantitative review evidence (Fuseini et al., 2022). Dignity therapy in palliative contexts has been beneficial for issues related to dignity, hope, and quality of life, although the impact varies among different outcomes and groups (Wulandari and Rochmawati, 2024). The main point is that maintaining dignity goes beyond simple kindness and involves structured actions that ensure a person remains visible in narrative, relational, and moral aspects.

Respectful care of older people includes asking before acting, explaining before touching, involving before deciding, maintaining privacy, avoiding infantilizing language, recognizing biography and allowing reasonable risk when risk is linked to meaning. The goal is not to idealize independence or ignore vulnerability, but to stop care from turning into a system that erases individuals. Within a self-worth framework, quality care not only ensures the safety of older individuals but also conveys that their preferences are still important.

#### 3.5.3 Social prescribing and community participation

By linking health systems to community-based sources of belonging, participation, and meaning, social prescribing presents a promising way to uphold self-worth. Its impact on self-worth is contingent upon its application. When social prescribing is merely about referring individuals to activities, it could become just another routine intervention. When viewed as a means to restore roles, mutual exchange, and social recognition, it can directly bolster the self-esteem system.

Social prescribing for older adults may lead to improvements in physical and psychosocial outcomes including social participation and well-being, but the evidence is heterogeneous and limited in terms of methodology (Percival et al., 2022). A wider systematic review of social prescribing services provided mixed evidence, with some studies reporting health, well-being, self-concepts, social contacts and daily functioning improvements but variable study quality (Pescheny et al., 2020). These results suggest a need for careful optimism instead of exaggeration. The self-worth systems framework clarifies the focus areas for social prescribing. A narrative review suggests that social prescribing and community involvement might enhance well-being in older adults by tackling loneliness, low mood, and diminished social connections (Bild and Pachana, 2022). Narrative and systematic review evidence suggests that social prescribing may improve some physical and psychosocial outcomes in older adults, including social participation and well-being, but the evidence remains heterogeneous and methodologically uneven (Pescheny et al., 2020; Bild and Pachana, 2022; Rapo et al., 2023). These elements align closely with maintaining self-worth: the intervention should determine what is important to the individual, link them to activities that have personal and social significance, and offer support without taking away their autonomy.

Thus, community involvement should be seen as more than just showing up. Engaging in a weekly activity might not maintain self-esteem if the elderly individual stays inactive, unnoticed, or on the sidelines. In contrast, when older adults participate in activities that involve teaching, organizing, mentoring, creating, volunteering, advising, or supporting others, it can help restore a sense of reciprocity and importance. Social prescribing should shift from asking ‘What activity can we refer this person to?’ to ‘What type of involvement would help this person feel acknowledged, valuable, and connected?’ This change is crucial for social prescribing to evolve into an intervention that preserves self-worth, rather than just managing loneliness.

#### 3.5.4 Intergenerational programs

Intergenerational programs link personal history with social contribution and future generations, which enhances self-worth. The feeling that experience does not count threatens self-worth in later life. By fostering situations where older adults pass on knowledge, give emotional backing, share expertise, partake in cultural traditions, and contribute to the development of younger people, intergenerational contact can counteract this.

Systematic reviews suggest that programs linking community-residing older adults with children could be beneficial, but the evidence varies in terms of quality and design (Peters et al., 2021). Previous reviews suggest that intergenerational programs can yield positive results for different generations, such as enhanced attitudes, social bonds, and psychosocial functioning (Canedo-García et al., 2017). Recent systematic reviews on exchange programs between adolescents and older adults also highlight benefits for both parties, while stressing the importance of clearer implementation and reporting standards (Webster et al., 2024). In terms of self-worth systems, the essential mechanism involves more than just contact between age groups; it is about reciprocal acknowledgment. Programs tend to maintain the self-esteem of older adults when they are engaged as partners, educators, narrators, mentors, and collaborators, rather than as passive recipients of attention from younger individuals. The same rule holds for younger participants: successful intergenerational activities should not treat them solely as tools to ‘assist the elderly.’ Both generations should form a relationship where they can both give and take.

Intergenerational initiatives might also help fight ageism by presenting older adults in roles that oppose stereotypes of decline, inflexibility, or lack of usefulness. Young people may alter their ageist views when they perceive older adults as competent, introspective, witty, imaginative, or emotionally giving. Concurrently, older adults might experience a revitalized sense of generativity and importance. Intergenerational programs can simultaneously impact personal, relational, and cultural aspects of the self-worth system. However, the evidence base remains limited by heterogeneity in program content, duration, participant selection, outcome measurement and follow-up. Future studies should test whether benefits arise from contact alone or from reciprocal roles that allow older adults to contribute, teach, advise and be recognized.

#### 3.5.5 Digital inclusion as self-worth protection

The focus on digital inclusion should be on boosting self-esteem, not just on providing technical skills. As services such as banking, transportation, healthcare, communication, and social engagement become more digital, lack of access to these systems can lead to reduced autonomy and heightened feelings of incompetence, dependency, and invisibility. Older individuals often experience digital exclusion not just as a struggle with technology, but as a sense that the world is evolving without them.

Research on measurement and intervention has started to shed light on this issue. A comprehensive review of how digital literacy is measured in older adults revealed that current tools differ significantly and frequently fail to adequately assess age-relevant digital skills (Oh et al., 2021). Research on digital health literacy interventions shows potential improvements in health status and management among the elderly (Dong et al., 2023). Qualitative data on older adults’ experiences with digital mental health tools underscores the significance of accessibility, trust, support, personalization, and perceived usefulness (Yin et al., 2024). The results imply that achieving digital inclusion goes beyond merely providing device access. It necessitates systems that older adults can rely on, comprehend, adjust to, and use without feeling embarrassed. A rapid review of interventions to address digital exclusion in older adults in the social care domain suggests that interventions may improve digital skills but also highlight the need to address structural barriers such as access, sustained support and context-specific design (Wale et al., 2025). Preserving self-respect depends on this. Teaching seniors to use digital tools is not enough if platforms are hard to access, authentication processes are strict, interfaces are too complex, or human options are removed. Digital inclusion should involve redesigning systems to ensure older adults can actively participate, rather than pushing them into systems that are not user-friendly.

Therefore, digital inclusion ought to adhere to principles based on dignity. Design interfaces to be easy to use without being childish, secure while remaining accessible, versatile without being perplexing, and encouraging without being patronizing. For critical services like healthcare, banking, and public administration, human backup should continue to be accessible. Training should focus on building relationships and confidence, rather than being corrective or based on shame. Within a self-worth systems framework, digital inclusion safeguards autonomy, social engagement, and dignity by preventing technological advancements from leading to social exclusion. The current evidence is stronger for improvements in digital skills, access and confidence than for direct effects on dignity or self-worth. Future digital inclusion research should therefore include outcomes such as perceived competence, autonomy, embarrassment, trust, social participation and continued access to non-digital alternatives.

## 4 Discussion

### 4.1 Main synthesis

This review proposed a later-life self-worth systems framework to explain how older adults’ perceived value is shaped, threatened and preserved in aging societies. The central synthesis is that self-worth in later life cannot be adequately understood as a stable individual trait or as a synonym for self-esteem. Rather, it is produced through the interaction of personal self-interpretations, relational recognition and structural arrangements. Autonomy, dignity, mattering, reciprocity, role continuity and social visibility are therefore not isolated psychosocial variables; they are interdependent dimensions through which older adults experience whether they remain valued persons under conditions of retirement, illness, dependency, bereavement, care transitions, digitalization and cultural change.

The reviewed evidence is strongest for adjacent literatures showing that self-esteem, loneliness, social relationships, purpose, ageism and social participation are associated with mental health, mortality, health behavior and cognitive outcomes. The evidence is weaker when the specific construct of later-life self-worth is treated as the direct mechanism. Therefore, the framework should be read as a state-of-the-art conceptual synthesis rather than as a claim that self-worth has already been established as an independent causal determinant of healthy longevity.

### 4.2 Engagement with controversies

Several controversies are central to this framework. First, the self-esteem literature continues to debate whether low self-esteem is primarily a vulnerability factor for later depression and anxiety or whether psychological distress produces a scar that lowers subsequent self-esteem. This debate matters because it cautions against assuming that self-worth is always the causal lever. In aging contexts, self-worth is likely to function both as a vulnerability factor and as an outcome of illness, exclusion, loss and social withdrawal. Second, the framework speaks directly to debates over successful aging. Classic successful aging models emphasized low disease and disability, high physical and cognitive functioning and sustained engagement with life. This approach helped move gerontology beyond purely deficit-based views of aging, but it has also been criticized for privileging independence, productivity and individual responsibility in ways that may stigmatize frailty, disability and dependency. The self-worth systems framework responds to this controversy by arguing that dependency is not the opposite of successful or healthy aging. The relevant threat is not dependency itself, but dependency organized through devaluation, loss of agency, loss of privacy and loss of social contribution. Third, the framework engages the tension between autonomy-centered and relational approaches to care. A narrow autonomy-centered approach may equate worth with independent choice, whereas overprotective care may preserve safety by limiting agency. Relational autonomy offers a more appropriate basis for later-life self-worth because it recognizes that agency can be supported through relationships, institutions and environments. This is especially important in long-term care, dementia care and family caregiving contexts. Fourth, evidence for many proposed interventions remains mixed. Social prescribing, intergenerational programs and digital inclusion are promising, but the available evidence often measures loneliness, participation, well-being or digital skills rather than self-worth itself. The framework therefore identifies plausible intervention mechanisms rather than claiming definitive intervention efficacy.

### 4.3 Theoretical contribution

The self-worth systems framework adds to existing theories in three ways. First, it reframes self-esteem as one signal of perceived social value rather than the full object of analysis. Second, it specifies six worth-relevant dimensions that are especially salient in later life: autonomy, dignity, mattering, reciprocity, role continuity and social visibility. Third, it emphasizes systemic feedback loops through which social conditions may amplify or repair perceived devaluation. For example, loneliness may lower self-worth, reduced self-worth may reduce social initiative and withdrawal may intensify loneliness. Digital exclusion may produce embarrassment, avoidance and further exclusion. Reciprocity may strengthen mattering, which may increase participation and reinforce social visibility. This systems perspective does not replace ecological, self-determination, sociometer, healthy aging or dignity-centered care theories. Rather, it integrates them around a more specific question: how do aging societies communicate to older adults that they remain valued, agentic and consequential?

### 4.4 Implications for research, practice and policy

For research, the immediate task is measurement. Later-life self-worth should not be reduced to global self-esteem, loneliness or life satisfaction. Measures should capture autonomy, dignity, mattering, reciprocity, role continuity, social visibility, cultural recognition and digital agency. Longitudinal and transition-focused designs are especially needed around retirement, bereavement, disability onset, hospitalization, entry into long-term care and adoption of digital services. Ecological momentary assessment could also capture within-person fluctuations in self-worth across daily social interactions and contexts (Shiffman et al., 2008). For practice, the framework suggests that interventions should not merely increase contact, safety or service access. They should restore meaningful agency and contribution. In care settings, this means asking before acting, preserving privacy, supporting reasonable risk, involving older adults in decisions and recognizing biography. In community settings, it means designing roles in which older adults are not only helped but also able to help, teach, advise, organize and participate. For policy, the framework suggests that healthy longevity should include psychosocial recognition as well as survival and function. Age-friendly environments, long-term care regulation, digital service design, retirement planning and public-health interventions should be assessed partly by whether they preserve older adults’ autonomy, dignity, contribution and social visibility.

### 4.5 Limitations of this review and future directions

This review has limitations. It is a narrative and conceptual review, not a systematic review or meta-analysis. The literature search was selective rather than exhaustive, and no formal risk-of-bias assessment was conducted. The framework also integrates evidence from adjacent constructs, including self-esteem, purpose, dignity, mattering, loneliness and social connection, because direct research on later-life self-worth systems remains limited. Biological embedding is particularly tentative and should be tested with stronger longitudinal and mechanistic designs. Cultural claims are also limited, especially where East Asian and Chinese examples are used to illustrate interdependent recognition systems. Future research should develop validated multidimensional measures of later-life self-worth, test the proposed feedback loops, evaluate whether interventions restore specific self-worth mechanisms and examine how cultural, socioeconomic, gendered and digital inequalities shape perceived worth. Mixed-method designs will be especially important because self-worth is both measurable and experiential: older adults must be asked not only whether interventions improve well-being, but whether they feel respected, needed, visible and able to matter.

## 5 Conclusion

Aging societies should not define healthy longevity only by survival, disease control or functional ability. Older adults also need conditions that allow them to remain recognized, agentic, connected and consequential. This review proposed a later-life self-worth systems framework organized around autonomy, dignity, mattering, reciprocity, role continuity and social visibility. The framework’s main contribution is to separate dependency from devaluation. Functional decline, care needs and digital difficulty do not automatically diminish self-worth. Self-worth is threatened when social systems respond to these changes by removing choice, privacy, contribution, recognition or visibility. Preserving self-worth therefore requires not only psychological support, but also better care practices, inclusive communities, age-sensitive technologies and policies that recognize older adults as continuing participants in social life.
